# Prognostic value of inflammation in prostate cancer progression and response to therapeutic: a critical review

**DOI:** 10.1186/s12950-016-0143-2

**Published:** 2016-11-25

**Authors:** Alessandro Sciarra, Alessandro Gentilucci, Stefano Salciccia, Federico Pierella, Flavio Del Bianco, Vincenzo Gentile, Ida Silvestri, Susanna Cattarino

**Affiliations:** 1 Department of Urology, University Sapienza of Rome, Rome, Italy; 2 Department of Molecular Medicine, University Sapienza of Rome, Rome, Italy; 3 Department of Urological science, University Sapienza, Viale Policlinico 155, 00161 Rome, Italy

**Keywords:** Prostate neoplasm, Inflammation, Radical prostatectomy, Radiotherapy, Hormone therapy

## Abstract

Prostate is an immune-competent organ normally populated by inflammatory cells. Prostatic inflammation origin can be multi-factorial and there are some emerging evidences on its possible role as a factor involved in prostate cancer (PC) pathogenesis and progression.

This review critically analyzes the role of inflammation as a prognostic factor for progression and aggressiveness of PC. We verified the last 10 years literature data on the association between inflammation and PC aggressiveness, or PC response to therapies.

Several studies tried to correlate different inflammatory factors with the aggressiveness and metastatization of PC; all data sustain the role of inflammation in PC progression but they also produce confusion to identify a reliable clinical prognostic marker.

Data on patients submitted to radical prostatectomy (RP) showed that cases with marked intraprostatic tissue inflammation are associated with higher rate of biochemical progression; systemic inflammation markers appear to have a significant prognostic value.

Analyzing data on patients submitted to radiotherapy (RT) emerges a significant association between high neuthrophil to lymphocyte ratio (NLR) and decreased progression free survival and overall survival; also plateled to lymphocyte ratio (PLR) and C-reactive protein (CRP) have been proposed as significant prognostic factors for progression and overall survival.

In patients submitted to androgen deprivation therapy (ADT), inflammation may drive castration resistant PC (CRPC) development by activation of STAT3 in PC cells. NLR has been proposed as independent predictor of overall survival in CRPC submitted to chemotherapy.

Most of data are focused on markers related to systemic inflammation such as NLR and CRP, more than specifically to chronic prostatic inflammation. The suggestion is that these inflammatory parameters, also if not specific for prostatic inflammation and possibly influenced by several factors other than PC, can integrate with established prognostic factors.

## Background

### Inflammation and prostate cancer: which evidences?

The prostate is an immune-competent organ and is normally populated by a small number of inflammatory cells [1]. In particular regulatory T-cells are mainly located in the fibromuscular stroma and in the peri-glandular area [2]. The origin of prostatic inflammation can be multifactorial, including bacterial infections, viruses, dietary factors, hormones, autoimmune responses and urine reflux [1, 3].

The persistence of some of these factors can constitute to chronic prostatic inflammation. Prostate cancer (PC) is now the most common cancer among men. The role of infection or inflammation is sustained in different cancer sites and also in PC. Epidemiologic, histo-pathologic and molecular pathologic studies provide the emerging evidence of the possible role of prostatic inflammation as a factor involved in PC initiation and/or progression [4–6]. Inflammation can play a role in PC carcinogenesis through several mechanisms: causing cellular and genomic damage; promoting cellular turnover; creating a tissue microenvironment inducing cell replication, angiogenesis and tissue repair [4, 7].

The high prevalence of chronic inflammation in pathological samples of prostate tissue from surgery or prostate biopsy has sustained a possible link between chronic inflammation and PC [8, 9]. Pro-inflammatory cytokines, inflammatory mediators and growth factors related to inflammation can determine uncontrolled proliferative response, rapidly dividing cells more likely to undergo mutation, as observed in cancer [7, 10]. Several PC susceptibility genes such as RNASEL, MSR1,MIC1 involved in PC carcinogenesis, encode proteins with functions in response to inflammation, infection and oxidative stress: their mutation may reduce the possibility of preventing carcinogenesis through these pathways [4, 11]. Some data show that chronic inflammation can induce proliferative events and post-translational DNA modifications in prostate tissue also through oxidative stress [8, 12]. Repeated tissue damage and oxidative stress related to this event may produce compensatory cellular proliferation with the risk of hyperplastic growth or neoplastic transformation [13]. It is accepted that prostatic inflammation can generate free radicals such as nitric oxide that can be converted by cyclo-oxigenase enzymes to different eicosanoids and prostaglandins that have been recognized as regulator of prostate cell proliferation [14]. Normally glutathione transferase activity defends prostate cells against the genomic damage induced by oxidants isolated at sites of inflammation [8]. The methylation of this gene produce the loss of his protective activity and it could be implicated in the translation from inflammation to pre-neoplastic lesions such as high grade prostatic intraepithelial neoplasia (PIN) and therefore to PC [14].

As clinical data, a 5-year follow-up study based on prostate biopsies established that chronic inflammation accounts for nearly 20% PC development [15, 16]. Moreover, inflammatory atrophy was found in about 40% of PC cores in that study. Observations from many lines of research indicate the existence of role of inflammation in prostate carcinogenesis and progression [17].

### Aim

The aim of the present review is to critically analyze the role of prostatic inflammation as a prognostic factor for the progression and aggressiveness of PC. In particular, we did not analyze the relationship between inflammation and PC development or incidence but, we verified data on the association between inflammation and PC aggressiveness, or PC response to therapies.

Our aim is to analyze whether, in cases with PC diagnosis, an association with an inflammatory status may determine a poorer prognosis, higher aggressiveness of the tumor and lower response to therapies. We also try to verify a possible role of prostatic inflammation as a prognostic factor in PC patients, for tumor progression despite surgical, radiotherapic or medical therapies.

### Methods

We searched in the Medline and Cochrane Library databases (primary fields: prostate neoplasm and inflammation; secondary fields: aggressiveness, Gleason score, prognostic, radical prostatectomy, radiation therapy, androgen deprivation therapy, CRPC without language restriction from the literature of the last 10 years. We included original articles, clinical trials conducted in humans and reviews.

Our review was divided in different sections analyzing the following items: relationship between inflammation and PC aggressiveness; influence of inflammation on the response to surgery, radiotherapy, androgen deprivation therapies; role of inflammation in CRPC. At the end of each section we critically analyzed data in terms of a possible prognostic role of prostatic inflammation in these specific settings.

## Results and discussion

### Inflammation and prostate cancer aggressiveness

Different clinical and pathological parameters and classifications are used to determine PC aggressiveness and risk of progression. In particular the Gleason grading is used in conjunction with other clinical and molecular variables, including serum prostate specific antigen (PSA) levels and clinical stage, to classify PC in risk classes of progression and to help make treatment decisions for PC.

PC is one of the most common causes of cancer-related deaths among males; however a significant percentage of diagnosed PC have a relatively indolent form that is unlikely to progress with time. A subset of PC, on the contrary, exhibit aggressive properties, such as rapid proliferation, metastatic spread, resistance to therapies. To obtain a correct management of PC, the major challenge remains to distinguish patients who need definitive treatments from cases who have a latent tumor.

Gurel et al. [18], histologically examined prostate biopsy samples for inflammation using the United States National Institute of Health consensus grading system. Cases with inflammation in ≥ 1 biopsy core were 1.78 times (95% CI 1.04–3.06) more likely to develop PC in the core examined in the biopsy than those who had no evidence of inflammation.

This association was more pronounced for high grade tumors (Gleason sum 7–10; OR 2.24, 95% CI 1.06–4.71) than for low grade cases (Gleason sum 6; OR 1.57,95% CI 0.83–300) [18].

From the placebo arm of the Prostate Cancer Prevention Trial, Gurel et al. [18] showed a significant association between the presence and extent of chronic inflammation in prostate biopsy cores and high grade PC (Gleason sum 7–10; OR-2.24;95% CI 1.06–4.71) but not in low grade cases (Gleason sum 6; OR 1.57; 95% CI 0.83–3.0).

### Adaptive immune cells

As adaptive immune cells, analysis of two major prostate tumor-infiltrating lymphocyte subsets showed that capsular and perineural invasion as well as biochemical progression was related to strong infiltration of T and B lymphocytes [15, 19]. Mc Ardle et al. [20] showed that the presence of increase CD4+ T-lymphocyte infiltrate was associated with poor PC prognosis and it can predict survival.

Ammirate et al. [21] found that PC progression and metastasis is associated with inflammatory infiltration of macrophages and lymphocytes and activation of I kappaB kinase (IKK) by an NF-kappaB independent mechanism.

Davidsson et al. [22] in a case control study on cases diagnosed for PC, showed that men with greater numbers of CD4+ regulatory lymphocytes in their PC environment have an increased risk of dying of PC and this identification may predict clinically relevant tumors.

Although the role of B-lymphocytes in cancer has been overshadowed by the interest in T-cell responses, B-cells can play a complementary role in the immune response against tumor [15]. Gannon et al. [23] compared metastatic and non-metastatic pelvic lymph nodes from PC cases and controls. They found a decreased presence of CD20+ B-lymphocytes and CD38+ lymphocytes in metastatic lymph nodes comparatively to control lymphnodes.

Recently neutrophil-to-lymphocyte ratio (NLR), an inflammatory parameter, has been reported to be of prognostic value in some solid tumors including PC [24]. Yin et al. [24] conducted a meta-analysis to obtain a reliable assessment of the prognostic significance of NLR calculated from the white blood cell differentiated counts in PC patients. A total of 14 studies were included and they showed that elevated NLR was not significantly associated with the poor overall survival (OS) (HR 1.45; 95% CI 0.77–2.71; *p* = 0.248) or recurrence-free survival (HR = 1.34; 95% CI 0.89–2.02; *p* = 0.155) of patients with localized PC. On the contrary elevated NLR predicted poorer OS (HR 1.57, 95% CI 1.41–1.74; *p* < 0.001) and progression-free survival (HR = 1.97;95% CI 1.28–3.04; *p* = 0.002) in metastatic PC. This finding was also confirmed in a second meta-analysis on 9418 PC patients from Tang et al. [25]. In the majority of studies, NLR was not significantly associated with Gleason grading in PC patients (*p* = 0.573) [25, 26].

### Innate immune cells

Various innate immune cells have been involved in PC progression. Macrophages are a source of secreted pro-inflammatory cytokines and they are distinguished in two phenotypes: M1 with tumor inhibitor properties and M2 with tumor stimulatory properties [15, 27, 28]. Lanciotti et al. [28] showed that a high density of macrophages in PC is associated with more aggressiveness and poorer prognosis. In particular M2 phenotype was more presented in Gleason score 8–10 and pT3a stage. As showed by Fang et al. [29] in co-cultured prostate epithelial cells, M2 induction of aggressiveness involved the alteration of signaling of macrophage, androgen receptor (AR), inflammatory chemokine CCL4-STATE3 activation, down regulation of p53/PTEN tumor suppressors.

### Chemokines

Besides immune cells, chemokine receptors have been shown highly expressed on tumor cells in comparison to normal glands [15] and evidences have been provided that chemokines are involved in the metastatization and progression of different tumors. In vivo based studies on chemokines in PC have been focused on CXCL8, CXCL12 and CCL2 [30–32]. Chemokines can facilitate tumor progression and dissemination either by shaping the functional profile of infiltrating lymphocytes or by activating stromal and neoplastic cells [15]. Changes in chemokine receptors may be necessary to activate tumor progressing signals. For example, more aggressive PC cells express higher levels of CCR2 compared with less aggressive cells and they increase also in metastatic compared to localized PC [33]. Moreover increased serum levels of CXCL8 and CCL2 have been associated as possible prognostic markers of a high grade and progressive PC [34, 35].

### Cytokines

Multiple inflammatory cytokines have been identified as potential mediators between prostatic inflammation and PC risk and aggressiveness [36]. One of these is macrophage inhibitory cytokine 1 (MIC-1), a member of the transforming growth factor beta family. Some evidence suggested an up regulation of MIC-1 related to PC aggressiveness and that circulating levels of MIC-1 can predict poor prognosis for PC [37]. IL-6 is a multifunctional cytokine which is expressed in most PC cell lines and the expression is at higher levels in those which do not express the androgen receptor (AR) and show an enhanced malignant potential [38]. Aberrant IL-6 signal transducer and activator of transcription-3 (STAT3) signalling and loss of p53 occur during PC progression to metastatic disease [39].

Neveu et al. [40], in primary prostate cell culture from patients submitted to radical prostatectomy, showed that the baseline IL-8 secretion level was associated with PC aggressiveness and the median baseline IL-8 levels were significantly lower in cases with non-aggressive PC compared to those having aggressive PC (*p* = 00005). In particular the Gleason score significantly increased with the basal level of IL-8 (rho =0.63; *p* = 0.0007).

Some studies reported on the association between one or multiple single nucleotide polymorphism (SNP) in inflammation-related pathways and prostate cancer risk and aggressiveness [36, 41]. Zabaleta et al. [42] evaluated 15 SNP in five cytokine genes (IL-1B,IL-10, TNF alfa, IL-6 and IL-8) in relation to risk of aggressive PC (Gleason score ≥ 8 and PSA > 20 ng/ml) and they found an association between aggressive PC and an IL-8 (IL-8-47CT) genotype (OR = 3.5;95% CI 1.13–10.88), as well as an increased risk with combined genotypes in IL-1B and IL-10 (OR = 3.38;95% CI 1.70–6.71).

SUMO-specific protease 1 (SENP1) is a member of the SUMOylation protease family and has a crucial role in the regulation of androgen receptor (AR) dependent transcription and inflammatory hypoxia signaling [43]. Wang et al. [43] analyzed 150 human PC specimens and they showed that SENP1 expression directly correlates with PC aggressiveness. In fact higher SENP1 scores corresponded with higher Gleason scores of PC patients. Moreover, silencing SENP1 level in metastatic PC cells, perturbs their ability to metastatize to the bone [43].

Fucosylation haptoglobin (Fuc-Hpt) is an important oligosaccharide modification associated with inflammation and cancer. Fujita et al. [44] in 98 PC cases submitted to radical prostatectomy showed that serum levels of Fuc-Hpt were significantly associated with Gleason score *P* < 0.01) and Fuc-Hpt levels were significantly higher in patients with Gleason score ≥ 7 than in those with Gleason score 6. Authors proposed Fuc-Hpt serum determination as a novel PC biomarker for predicting aggressiveness and prognosis [44].

Another important cytokine involved in PC progression appears to be the macrophage migration inhibitory factor (MIF) [45]. Tawdros et al. [46] showed that an increased extracellular release in MIF induces neuroendrocrine (NED) differentiation in PC stimulating an AR independent progression. The relevance of this study is to link MIF release in chronic prostatic inflammation, to the development of an aggressive phenotype such as NED in PC.

Recently, an emerging class of inflammasomes is considered as master regulators of inflammation. Inflammasomes are a group of multimeric proteins that consist of NLR protein, an apoptosis-associated speck-like protein containing a carboxyterminal CARD (ASC), and procaspase-1 [47]. Assembly of inflammasomes complex activates caspase-1 leading to the secretion and the maturation of proinflammatory cytokines IL-1 and IL-18, which causes a wide variety of biological effects associated with infections. NLRP3 is one of the most studied inflammasomes and senses pathogens and danger signals in response to injury or infection. Therefore, various stimuli such as urine reflux, uric acid crystals, bacteria, or fungi may lead to activation of inflammasome-medicated proinflammatory cytokines in the prostate driving tumor development [47].

### Mast cells

MCs (MCs) are recognized as important effectors in Immunoglobulin-E (Ig-E) associated immune responses, and potent effector cells of the innate immune system [48]. In some tumor settings MCs have a protective role, exerted by their proinflammatory mediators, while in other tumors MCs may directly influence the advancement of the cancer cells by stimulating the neovascularity, tissue remodeling and modulation of the host immune response [48]. In PC, MCs have been only recently indicated as potential independent prognostic marker and MCs targeting associated with castration suggested as a potential therapy. Xie et al., in vitro and in vivo models, [49] demonstrated that PCa cells may have better capacity than normal prostate to recruit mast cells and infiltrating mast cells could enhance PCa resistance to chemotherapy and radiotherapy via activation of p38/p53/p21 and ATM protein kinase signals.

### Critical analysis

Several studies in the literature tried to correlate different inflammatory factors with the aggressiveness and metastatization of PC. Innate and adaptive immune cells, chemokines and cytokines, all showed to significantly correlate to PC aggressiveness (Table 1). These data together confirm the role of inflammation in PC progression but they also produce confusion to identify a reliable clinical prognostic marker. However, innate and adaptive immunity cells may play a dichotomous role, acting in the context of both pro-tumorigenic and anti-tumorigenic depending on the stage of disease, type of inflammation, and/or the tumor microenvironment [6].

**Table 1:** OR and 95% CI values for the association between different inflammatory parameters and PC aggressiveness

Parameter	Reference	Study characteristics	Association	OR (95% CI)	*P* value
Histological prostatic inflammation	[17]	Phase II study on prostate biopsies	Inflammation and high grade PC	2.24 (1.06–4.71)	<0.01
Histological prostatic inflammation	[17]	Prostate cancer prevention trial on prostate biopsy	Inflammation and high grade (Gleason score 7–10) PC	2.24 (1.06–4.71)	<0.01
Serum NLR	[26]	metanalysis	NLR and high grade PC	–	0.573
Serum SNP	[41]	Phase II study	IL-8 and high grade PC	3.5 (1.13–10.88)	<0.01
Serum SNP	[41]	Phase II study	IL-1B and IL-10	3.38 (1.70–6.71)	<0.01

Some of these factors can be detected only on prostate tissue samples, others have been suggested as serum markers. NLR can be simply obtained in the clinical practice and it is presented in several studies as a strong indicator of PC prognosis and risk of progression in metastatic cases. Its role seems to be independent to the Gleason score of the tumor.

Among Inflammatory cytokines, MIC-1, IL-6 and IL-8 may be prognostic markers of aggressiveness and metastatic progression but data on their serum determination remain limited. MCs have been recently indicated as novel independent prognostic markers in PC, although previous studies on prostatic biopsies associated high MC densities with favorable tumor characteristics and good prognosis. An explanation for these appearing to be a discrepant finding remains the observation that PC is a multifocal and heterogeneous disease [48, 49].

The potential mechanistic relationships between the molecular events associated with the persistent inflammatory response and prostate carcinogenesis have important implications for optimizing the current therapies against different prostatic disorders and PC [50].

## Prognostic value of inflammation in prostate cancer submitted to radical prostatectomy

Radical prostatectomy (RP) is the main treatment choice for localized PC. Risk of biochemical and clinical progression is related to different clinical parameters such as Gleason score, preoperative PSA, pathological staging. Currently, RP is the only treatment for localized PC able to show a benefit for OS and cancer-specific survival (CSS), compared with conservative management, as shown in one prospective randomized trial [51]. Preoperative models for PC risk stratification include D’Amico or Partin tables and they are based on initial PSA values, biopsy Gleason score and clinical T stage.

Klink et al. [52] analyzed 287 men with PC submitted to RP to explore the association between tumor inflammation, adverse pathology and biochemical progression after surgery. Mild and marked inflammation was found in 53 and 30% of tissue samples respectively. Cases with marked inflammation were significantly more likely to have higher clinical stage, higher PSA, higher percent of cores positive and positive margins. On univariate analysis, increasing inflammation grade was associated with progressively higher risk of positive margins (OR 2.26–3.56), capsular penetration (OR 3.12–3.19), seminal vesicle invasion (OR 4.08–6.83). After adjusting for multiple clinical characteristics, higher grade of inflammation remained significantly associated with risk of capsular penetration, positive margins and seminal vesicle invasion. Overall, increasing inflammation grade was associated with biochemical progression (*p* = 0.02). Mild and marked inflammation were associated with 2.18 (95% CI 1.16–4.14) and 2.55 (95% CI 1.31–4.97)—fold greater biochemical progression rate versus no inflammation. However the association of inflammation with biochemical progression rate did not reach statistical significance (*P* > 0.2) after adjustment with the other clinical parameters.

Systemic inflammation is a host reaction to carcinogenesis or cancer progression and serum levels of butyrylcholinesterase (BChE) have been reported to reflect the presence of inflammation and other clinical conditions [53]. Serum levels of BChE has been found to decrease during inflammation. Koie et al. [54] in a retrospective study reviewed the pathological records of 535 PC patients submitted to RP. The cut-off point for the serum BChE level was determined as 168 IU/L according to previously described methods. Biochemical progression free survival rates were 77.7% in BChE ≥ 168 IU/L (95% CI 115.2–152.2) and 55.0% in BChE < 168 IU/L (95% CI 104.4–131.7; *p* < 0.001). On multivariate analysis, initial PSA and serum BChE levels were independent significant predictors of biochemical progression.

Shafique et al. [55] investigated the role of inflammation-based prognostic scores, the modified Glasgow Prognostic Score (mGPS) and neutrophil-lymphocyte ratio (NLR), to predict progression after RP. The mGPS was constructed by combining C-reactive protein and albumin whereas NLR by calculating the ratio of neutrophils to lymphocytes. Systemic inflammation appeared to have significant prognostic value. The mGPS predicted poorer 5-year overall survival independent to age, PC grade and NLR. Raised mGPS had a significant association with excess risk of death (OR 2.41; 95% CI 1.37–4.23) among aggressive, clinical significant PC.

### Critical analysis

Data on a possible prognostic role of prostatic inflammation in the clinical response to RP in PC are limited in the literature (Table 2). As of now, the studies are mainly retrospective, but some findings may suggest that the grade of inflammation in preoperative biopsy specimens could be used to risk-stratify men with PC for risk of biochemical progression after RP [54, 55]. However, in a multivariate analysis after adjustment with the other clinical parameters (PSA, clinical stage, Gleason score), inflammation did not result an independent predictor. The suggestion that serum markers of systemic inflammation may be independently related to the biochemical progression free survival must be confirmed in prospective studies.

**Table 2:** OR and 95% CI values of different inflammatory parameters used as prognostic indicators for PC submitted to radical prostatectomy

Parameter	Reference	Study characteristics	Association	OR (95% CI)	*P* value
High grade histological prostatic inflammation	[47]	Phase II study on RP	High grade inflammation and positive surgical margins or capsular penetration or seminal vesicle invasion	2.26–3.56	<0.01
				3.12–3.19	
				4.08–6.83	
High grade histological prostatic inflammation	[47]	Phase II study on RP	High grade inflammation and biochemical recurrence	2.55 (1.31–4.97)	0.02
Systemic inflammation on mGPS	[50]	Phase II study on RP	mGPS and risk of death	2.41 (1.37–4.23)	<0.01

## Prognostic value of inflammation in prostate cancer treated by radiotherapy

Definitive radiotherapy is a treatment option for localized or locally advanced PC. Clinical parameters related to treatment response and risk of progression are serum PSA, clinical T stage and biopsy Gleason score.

The results of the Glasgow Inflammation Outcome Study [56, 57] demonstrated that an increased neuthrophil-to-lymphocyte ratio (NLR) had a prognostic value in localized PC. However the study included a rather heterogeneous population of PC patients. Langsenlehner et al. [58] analyzed 415 consecutive patients with histologically confirmed PC who underwent radiation therapy. Based on previous data, a NLR cutoff of 5 was applied to differentiate between low (<5) and high (≥5) NLR and it was evaluated before the radiotherapy. A significant association between high NLR and decreased progression free survival (*p* = 0.015) and decreased overall survival (*p* = 0.011) was found. Both univariate and multivariate analysis showed that elevated NLR was associated with decreased clinical progression free survival (HR 3.09; 95% CI 1.64–5.82 *p* < 0.001) and decreased overall survival (HR 2.16; 95% CI 1.17–3.99; *p* = 0.013). The prognostic value of NLR remained significant also after adjustment for other clinical parameters such as Gleason score and clinical stage. Bahig et al. [59] retrospectively analyzed 950 PC cases submitted to radiotherapy for localized disease. Neutrophil count (median 4.5 × 10^3^/μl, range 0.9–12) (HR =1.18;95% CI 1.01–1.37,*p* = 0.028) but not NLR (median 3.0, range 0.1–18.4) was associated to overall survival.

Also plateled-to-lymphocyte ratio (PLR) which is calculated as the ratio of plateled count to lymphocyte count has been proposed as an assessable marker of systemic inflammation and has been presented as prognostic marker of different cancers. Langsenlehner et al. [60] evaluated 374 PC patients treated with external 3D radiotherapy. The optimal cut-off level for PLR was fixed at 190. A high PLR was a significant prognostic factor for metastasis free survival (HR = 2.24 95% CI 1.06–4.76; *p* = 0.036), cancer specific survival (HR 3.99;95% CI 1.19–13.4; *p* = 0.025) and overall survival (HR 1.87;95% CI 1.02–3.42; *p* = 0.044) by multivariate analysis. The prognostic value of PLR remained significant and independent also after adjustment for Gleason score and clinical stage.

Schoenfeld et al. [61] evaluated association between single nucleotide polymorphisms (SNP) in RNASEL, a gene implicated in inflammation and clinical response of 434 PC cases submitted to radiotherapy. SNP of rs12757998 was associated with a significant decreased risk for biochemical recurrence (HR 0.60; 95% CI 0.40–0.89; *p* = 0.02). This association remained significant also after adjustment for Gleason score and clinical stage, or selecting only cases at high risk.

Thurner et al. [62] in 261 PC patients treated with external 3D conformational radiotherapy, analyzed the prognostic relevance of elevated plasma C-reactive protein (CRP) as a marker of inflammation. The optimal cut-off level for CRP was 8.6 mg/l. An elevated CRP plasma level was associated with decreased cancer specific survival (HR 3.36; 95% CI 1.42–7.91; *p* = 0.006) and overall survival (HR 3.24;95% CI 1.84–5.71; *p* < 0.001) at univariate and multivariate analysis. No significant association between CRP and Gleason score or tumor stage was found (*p* > 0.05). Using ROC curve analysis, the authors calculated ideal cut-off values of CRP in the three risk groups at 8.9,8.4 and 13.4 respectively.

### Critical analysis

More clinical data on the relationship between inflammation and response to radiotherapy for PC are present in the literature (Table 3). The populations analyzed are larger and more homogeneous and data are examined by multivariate analysis so to define their independent prognostic value. Markers of systemic inflammation more than marker specific for prostatic inflammation have been examined. In particular NLR but also PLR and CRP showed a significant and independent prognostic value for patients submitted to radiotherapy, either in terms of clinical progression or of overall survival, also after adjustment for Gleason score and clinical stage [59, 60]. In the intermediate and high risk classes as described by D’Amico et al., NLR and CRP are able to furthermore stratify cases in terms of clinical progression free survival [62].

**Table 3:** OR and 95% CI values of different inflammatory parameters used as prognostic indicators for PC submitted to radiotherapy

Parameter	Reference	Study characteristics	Association	OR (95% CI)	*P* value
Systemic inflammation NLR	[53]	Phase II on radiotherapy as primary treatment	NLR and clinical progression free survival or overall survival	3.09 (1.64–5.82)	<0.001
				2.16 (1.17–3.99)	0.013
Systemic inflammation PLR	[55]	Phase II study on radiotherapy as primary treatment	PLR and metastasis free survival or cancer specific survival or overall survival	2.24 (1.06–4.76)	0.036
				3.99 (1.19–13.4)	0.025
				1.87 (1.02–3.42)	0.044
SNP in RNASEL	[56]	Phase II study on radiotherapy as primary treatment	SNP of rs12757998 and biochemical recurrence	0.60 (0.40–0.89)	0.02
Systemic inflammation CRP	[57]	Phase II study on radiotherapy as primary treatment	CRP and cancer specific survival or overall survival	3.36 (1.42–7.91)	0.006
				3.24 (1.84–5.71)	<0.001

Elevated NLR and CRP levels may reflect increased concentrations of these pro-inflammatory cytokines that create a microenvironment favouring PC proliferation and metastatization despite radiotherapy [62]. Furthermore, raised CRP concentrations may produces increased serum levels of vascular endothelial growth factor (VEGF) [63, 64]. However NLR, PLR and CRP are not specific markers of inflammation that might be influenced by several other conditions such as active infection, inflammatory diseases and smoking behaviour independent of PC.

## Prognostic value of inflammation in prostate cancer treated by androgen deprivation therapy and in castration resistant PC

Androgen deprivation therapy (ADT) can be achieved by either suppressing the secretion of testicular androgens using a surgical castration via orchiectomy or a medical castration via inhibiting the hypothalamic pituitary axis using LH-RH agonists or antagonists or inhibiting the action of circulating androgens at the level of their receptor using competing compounds known as anti-androgens. In addition, these two methods can be combined to achieve what is known as complete (or maximal or total) androgen blockade (CAB).

An in vivo model demonstrated that during ADT infiltrated of B cells can activate NFkB kinase with the up-regulation of cytokines such as CXCL-1,IL-8,IL-12,TNF alpha. These cytokines activates STAT3 in PC cells and shortens the duration of ADT response promoting tumor cell survival through anti-apoptotic signaling [21]. These treatments can be involved in the development of a CRPC [65].

Sharma et al. [66] analyzed 122 metastatic PC cases treated by ADT and measured in the serum different cytokines such as MCP-1,IL-1,IL-2,IL-6,IL-8 and TNF alpha. On multivariate analysis only IL-8 (HR 1.9;95% CI 1.0–3.5, *p* = 0.04) and TNF alpha (HR 2.0;95% CI 1.1–3.5; *p* = 0.02) resulted significant and independent predictors of overall survival during ADT.

Treatment options for CRPC have expanded with the introduction of several new approved agents including abiraterone, enzalutamide, radium 233 and different chemotherapic agents. Docetaxel with prednisone remain the standard first-line chemotherapy for treatment of CRPC. Prognostic and predictive biomarkers for response to the different agents are urgently needed so to identify the better therapeutic sequences for each patient. Inflammation in the prostatic microenvironment may also drive CRPC development during ADT.

Templeton et al. [67] analyzed in 356 metastatic CRPC patients submitted to docetaxel the prognostic role of NLR. In univariate analysis higher performance status, LDH and NLR (HR 1.65, 95% CI 1.25–2.18; *p* < 0.001) at baseline were associated with shorter OS. In multivariate analysis the highest statistical significance was obtained when liver metastases, hemoglobin <120 g/L, LDH > 1.2 X ULN and NLR > 3.0 were included in the model.

Similarly Lorente et al. [68] analyzed the prognostic role of NLR in a phase III study on 755 metastatic CRPC patients submitted to cabazitaxel as second line chemotherapy. Baseline NLR was associated with OS in univariate (HR 2.89;95% CI 2.12–3.94; *p* < 0.001) and multivariate analysis (HR 1.91;95% CI 1.31–2.79; *p* = 0.001) also adjusted for other clinical parameters. Authors selected a NLR cut-off of 3 as the most appropriate. Patients with a <3 NLR level had a statistically significant higher median OS (15.9 months versus 12.6 months, HR 1.55;95% CI 1.3–1.84; *p* < 0.001). than patients with a NLR ≥ 3.

Van Soest et al. [69] used data from two randomized phase III studies on 2230 men with metastatic CRPC patients submitted to docetaxel, to investigate the prognostic value of NLR. The optimal threshold of NLR for the prediction of OS was set a 2.05. In multivariate analysis both NLR and ADT duration before CRPC patients were associated with an increased risk of death (NLR HR 1.29;95% CI 1.11–1.50; *p* < 0.001) after adjustment for the other clinical parameters. Survival curves showed an OS of 26 months (95% CI 23.8–27.8) for men with a NLR < 2.0 and 19.1 months (95% CI 17.7-20.2) for men with a NLR ≥ 2.0. Sonpavde et al. [70] evaluated the prognostic impact of NLR as a marker for inflammatory and immune state in men with mCRPC following docetaxel. It is shown that NLR may be associated with an independent poor prognostic impact in post-docetaxel patients with mCRPC (1.55 [1.32, 1.83], *P* < .001). In particular NLR determination increased the c-statistic from 0.703 to 0.715 in a prognostic model that included LDH levels, hemoglobin, > 1 organ involved by metastatic disease, alkaline phosphatase, the number of prior cycles of docetaxel and progression to docetaxel.

Liao et al. [71], in 115 metastatic CRPC patients submitted to docetaxel, showed that median progression free survival (9.8 versus 7.5 months; *p* < 0.001) and OS (26.5 versus 13.5 months; *p* = 0.002) were higher in cases who did not have an elevated CRP than in those with an elevated CRP. In particular in a multivariate analysis, patients with a CRP > 8 mg/l were at significantly higher risk of tumor progression (HR 2.18; 95% CI 1.40–3.40; *p* = 0.001) and death (HR 2.0; 95% CI 1.28–3.12; *p* = 0.002) than patients with a CRP ≤ 8 mg/l.

Expanding on the utility of a single biomarker, the modified Glasgow Prognostic Score (mGPS) calculated using CRP and albumin levels, has been identified as a powerful marker of systemic inflammation [72]. Linton et al. analyzed the prognostic value of mGPS and NLR in 220 metastatic CRPC chemotherapy naïve patients but considered for docetaxel treatment [72]. NLR was not significantly prognostic for OS (HR 0.98; *p* = 0.91). On the contrary albumin (HR 0.28; 95% CI 0.14–0.56; *p* < 0.01) and CRP (HR 1.22; 95% CI 1.0–1.48; *p* = 0.048) were independently prognostic for OS. Combining the two parameters, a statistically significant association (*p* < 0.001) was observed between mGPS and lower OS (HR 1.87; 95% CI 1.35–2.59) with a marked decline in survival at higher scores (median survival 23.5 months at mGPS 0; 9.8 months at mGPS 2).

Leibowits-Amit et al. [73], in 116 metastatic CRPC patients treated with abiraterone, showed that NLR is a significant prognostic factor (OR 0.8; 95% CI 0.6–0.9,*p* = 0.05) for prediction of PSA progression. Using a NLR cut-off of 5, at multivariate analysis, a NLR ≤ 5 associated with restricted metastatic spread remained significantly predictor for PSA progression (OR 4.3; 95% CI 1.4–13.3; *p* = 0.01).

### Critical analysis

Systemic inflammation represents a truly novel and unexplored prognostic variables in CRPC [74], independent to other clinical parameters such as stage. It does not substitute but rather integrates with established prognostic factors. Pinato et al. [74] suggested that inflammation may predict not only survival but also cancer-related symptomatic burden (association between NLR and performance status) and risk of chemotherapy-related toxicity (inflammation-related alterations in drug pharmacokinetic).

Data on CRPC are strongly significant and are obtained in large populations from randomized trials. Different phase II and III trials [66–70] used OS and clinical progression free survival as end-points to evaluate the prognostic role of inflammatory variables (Table 4).

**Table 4:** OR and 95% CI values of different inflammatory parameters used as prognostic indicators in metastatic PC submitted to ADT or in metastatic CRPC

Parameter	Reference	Study characteristics	Association	OR (95% CI)	*P* value
Serum levels IL-8	[61]	Phase II study in metastatic PC submitted to ADT	IL-8 and overall survival or TNF alpha and overall survival	1.9 (1.0–3.5)	0.04
				2.0 (1.1–3.5)	0.02
Systemic inflammation NLR	[62]	Phase II study on CRPC submitted to docetaxel	NLR and overall survival	1.65 (1.25–2.18)	<0.001
Systemic inflammation and NLR	[63]	Phase II study on CRPC submitted to carbazitaxel	NLR and overall survival	1.91 (1.31–2.79)	0.001
Systemic inflammation NLR	[64]	Phase III study on CRPC submitted to docetaxel	NLR and risk of death	1.29 (1.11–1.50)	<0.001
Systemic inflammation NLR	[65]	Phase II study on CRPC submitted to sunitinib	NLR and overall survival	1.55 (1.32–1.83)	<0.01
Systemic inflammation NLR	[65]	Phase II study on CRPC submitted to abiraterone	NLR and PSA progression	4.3 (1.4–13.3)	0.01
Systemic inflammation CRP	[66]	Phase II study on CRPC submitted to docetaxel	CRP and risk of tumor progression or risk of death	2.18 (1.40–3.40)	0.001
				2.0 (1.28–3.12)	0.002
Systemic inflammation mGPS	[67]	Phase II study on CRPC submitted to docetaxel	mGPS and overall survival	1.87 (1.35–2.59)	<0.001

Both NLR and CRP resulted independent and significant predictors of OS and progression free survival in CRPC cases during chemotherapy with Docetaxel and for NLR also during abiraterone . An optimal threshold of NLR for the prediction of OS was set at 2–3 whereas of CRP at 8 [71].

Externally validated prognostic algorithms, in patients with metastatic CRPC considered for docetaxel, including NLR or CRP have been proposed [66–70].

The results of these studies qualify the NLR as an independent predictor of mortality in CRPC patients and support its incorporation in prognostic models to stratify patients with CRPC.

## Conclusions

The high prevalence of chronic inflammation in pathological samples of prostate tissue from surgery or prostate biopsy has sustained a possible link between inflammation and PC. In cases with PC diagnosis, an association with an inflammatory status may determine a poorer prognosis, higher aggressiveness of the tumor and lower response to therapies. These aspects have been analyzed in several clinical trials; however the majority of data are retrospective rather than prospective. Specific markers of prostatic inflammation have not been significantly identified as possible prognostic predictors.

On the contrary, most of data are focused on markers of systemic inflammation. in particular NLR and CRP, that can be easily obtained from the serum. Based on multivariate analysis they resulted independent significant predictors of clinical response to radiotherapy in localized and locally advanced PC or chemotherapy in CRPC cases, also after adjustment for the other clinical parameters. The suggestion is that these inflammatory parameters, also if not specific for prostatic inflammation and possibly influenced by several factors other than PC, can integrate with established prognostic factors. Validated prognostic algorithms including NLR or CRP have been proposed in particular for CRPC cases but prospective clinical trials should confirm these positive data.

## Abbreviations

ADT: Androgen deprivation therapy
AR: Androgen receptor
CRP: C-reactive protein
CRPC: Castration resistant PC
CSS: Cancer-specific survival
Fuc-Hpt: Fucosylation haptoglobin
mGPS: modified Glasgow Prognostic Score
MIF: Migration inhibitory factor
NLR: Neuthrophil to lymphocyte ratio
OS: Overall survival
PC: Prostate cancer
PIA: Prostatic inflammatory atrophy
PIN: Prostatic intraepithelial neoplasia
PLR: Plateled to lymphocyte ratio
PSA: Prostate specific antigen
PSA: Prostate specific antigen
RP: Radical prostatectomy
RT: Radiotherapy
SENP1: SUMO-specific protease 1
